# Vaccine promotion strategies in community pharmacy addressing vulnerable populations: a scoping review

**DOI:** 10.1186/s12889-023-16601-y

**Published:** 2023-09-23

**Authors:** Alexandre Chadi, Daniel J. G. Thirion, Pierre-Marie David

**Affiliations:** 1 Faculty of Pharmacy, Université de Montréal, Montreal, QC Canada; 2 McGill University Health Centre, Montreal, QC Canada

**Keywords:** Vaccination, Pharmacy, Healthcare disparities, Vulnerable populations, Promotional strategies

## Abstract

**Context:**

Social determinants of health are drivers of vaccine inequity and lead to higher risks of complications from infectious diseases in under vaccinated communities. In many countries, pharmacists have gained the rights to prescribe and administer vaccines, which contributes to improving vaccination rates. However, little is known on how they define and target vulnerable communities.

**Objective:**

The purpose of this study is to describe how vulnerable communities are targeted in community pharmacies.

**Methods:**

We performed a systematic search of the Embase and MEDLINE database in August 2021 inspired by the Preferred Reporting Items for Systematic Reviews and Meta-Analyses protocols (PRISMA ScR). Articles in English, French or Spanish addressing any vaccine in a community pharmacy context and that target a population defined as vulnerable were screened for inclusion.

**Results:**

A total of 1039 articles were identified through the initial search, and 63 articles met the inclusion criteria. Most of the literature originated from North America (*n* = 54, 86%) and addressed influenza (*n* = 29, 46%), pneumococcal (*n* = 14, 22%), herpes zoster (*n* = 14, 22%) or human papilloma virus vaccination (*n* = 14, 22%). Lifecycle vulnerabilities (*n* = 48, 76%) such as age and pregnancy were most often used to target vulnerable patients followed by clinical factors (*n *= 18, 29%), socio-economical determinants (*n* = 16, 25%) and geographical vulnerabilities (*n *= 7, 11%). The most frequently listed strategy was providing a strong recommendation for vaccination, promotional posters in pharmacy, distributing leaflet/bag stuffers and providing staff training. A total of 24 barriers and 25 facilitators were identified. The main barriers associated to each vulnerable category were associated to effective promotional strategies to overcome them.

**Conclusion:**

Pharmacists prioritize lifecycle and clinical vulnerability at the expense of narrowing down the definition of vulnerability. Some vulnerable groups are also under targeted in pharmacies. A wide variety of promotional strategies are available to pharmacies to overcome the specific barriers experienced by various groups.

**Supplementary Information:**

The online version contains supplementary material available at 10.1186/s12889-023-16601-y.

## Introduction

The COVID-19 pandemic has shed light on vaccination discrepancy between and within countries as we had both the technical and financial means to vaccinate individuals of every country [1]. It is estimated that 234,00 deaths could have been prevented in the US between June 2021 and March 2022 with a primary series of vaccinations [2]. Low vaccination rates disproportionately affect communities commonly defined as “vulnerable”. According to the Center for Disease Control and Prevention, infants from families with income below the poverty line are 30% less likely to receive the 7 recommended vaccines (measle-mumps-rubella, diphtheria-pertussis-tetanus, polio) for children aged 19–35 months [3]. Revenue is not the only factor influencing access to vaccination. Vaccination underservice directly affects communities’ health; as Black, Indigenous and Hispanic individuals in comparison with non-Hispanic White individuals have higher influenza-related hospitalization rates [4]. Population health is directly linked to the upstream societal structures and institutions that shape communities, to the relationship between individuals and to health seeking behaviours [5]. Vulnerability to infectious diseases can be associated to individual characteristics (e.g. age, pregnancy, disease state, disability), to habits (e.g. sexual practices, use of alcohol, illicit drug use, travelling) or to wider determinants such as social status, physical environment or social support [6].

In recent years, vaccination in community pharmacies is gaining momentum and may present a solution to reduce vaccine disparity. Pharmacists are recognized as accessible, convenient, trustworthy and cost-effective vaccine providers [7–10]. Studies from various jurisdictions show that allowing pharmacists to vaccinate increases uptake [11–14]. Pharmacies have surpassed medical offices in the provision of flu vaccines in the United States and in Canada [15, 16]. Prior reviews have focused on vaccine acceptability, accessibility and vaccine uptake following policy to allow pharmacists as immunizers [10, 17–20]. To our knowledge, no review has been conducted on how pharmacists reach eligible communities. Pharmacies are privately owned businesses and although pharmacists are dedicated to the well-being of their patients, some commercial practices may not be aligned with public health objectives of reaching those who need it the most. Certain pharmacies seem to adopt proactive methods to target vulnerable communities while others may rely on a ‘’first come first serve basis’’ [21]. As key contributors to vaccination, pharmacists must revise their implicit and explicit assumptions since it impacts how they define and reach vulnerable populations through their vaccine services [22]. Indeed, public health research has shown that “vulnerable populations” are not fixed identities, but the result of a process, which should be questioned from the perspective of vaccine services delivery in community pharmacies.

Evidence on the characteristics of patients vaccinated in pharmacy settings suggests that pharmacies vaccinate a more privileged population during influenza mass campaigns. Pharmacies tend to vaccinate individuals with a higher income [23, 24], higher education [24, 25] and younger populations [14]. Other traits such as being immigrant [23, 26], having diabetes or hypertension [23] and having a high number of chronic diseases [25] meant individuals were more likely to obtain their vaccine in a physician’s office. As definitions of vulnerable populations are multiple and go beyond clinical condition factors, understanding what characteristics pharmacists perceive as vulnerable remains key. The perception of vulnerability trickles down into how pharmacies target vulnerable populations, when they do, and may help finding solutions to vaccine discrepancy.

### Objective

Our objective is therefore to describe how vulnerability is defined and how vulnerable communities are targeted in community pharmacies within the published literature. More precisely, we seek to meet the following 4 objectives:

1. Describe the studies on the vaccination of vulnerable communities in pharmacies;
2. Identify which vulnerability characteristics are used to target underserved communities;
3. Document the barriers and facilitators towards vaccinating vulnerable communities in pharmacies;
4. Discuss which strategies are used by pharmacists and their team to target vulnerable communities.

## Methods

Based on the framework of scoping studies, our work seeks to describe, identify and synthesize the gaps in the existing literature [27]. Scoping reviews are useful to map out the existing literature on newer topics and orient future research. In our case, this review will allow us to better understand how pharmacists conceive vulnerability and how it impacts their implicit or explicit actions to address vaccine discrepancies. This scoping review will also determine the value of undertaking a full systematic review. We followed the checklist from the Preferred Reporting Items for Systematic reviews and Meta-Analyses extension for Scoping Reviews (PRISMA- ScR) [27]. With the help of an experienced librarian, we identified the relevant keywords and MeSH on the following 3 topics: ‘vaccination’, ‘pharmacy’ and ‘vulnerable populations’. The search strategy was then elaborated for the Embase database and adapted according to the MEDLINE database.

### Search strategy

The search was performed on 16 August 2021 to identify all articles in English, French or Spanish addressing vaccination of vulnerable communities in a pharmacy setting. We chose not to limit our study to a specific time period. This allowed gathering data from countries or states at different legislative stages regarding the status of pharmacists as prescribers and vaccinators. Inclusion criteria for this scoping review are the following: a) articles in a community pharmacy setting; b) articles where vaccination targets a population defined as vulnerable to an illness targeted by the vaccine; and c) peer-reviewed quantitative or qualitative studies or reviews studies.

Exclusion criteria are: a) articles providing insufficient details on vaccination in a pharmacy setting; b) vaccination by a pharmacist that occurs outside of a community pharmacy setting; c) articles where vaccination was not the primary focus; d) vaccine guidelines for healthcare professionals.

### Data extraction

Articles were imported into Zotero for duplicate removal, initial screening of titles and abstracts. The main author and one coauthor independently screened the initial 100 articles to reach a kappa consensus coefficient above 80%. Discrepancies were resolved through consensus. The remaining articles were sorted by the main author and uncertain articles were debated with a co-author. For feasibility purposes, data were extracted by one member with the use of a grid validated by co-authors.

The data extracted are comprised of the publication year, the methodology, the study population, the target strategy, the outcomes, and the barriers and facilitators reported by the authors. Results were then compiled, and descriptive statistics were generated through Excel software. The various target strategies were classified according to emerging categorization of passive, active and indirect promotion tactics. We finally identified the specific barriers to each vulnerable group and matched them with promotional strategies that overcome them. A quality of appraisal was not undertaken due to the anticipated heterogeneity of studies.

## Results

### Article overview

**Fig. 1:**
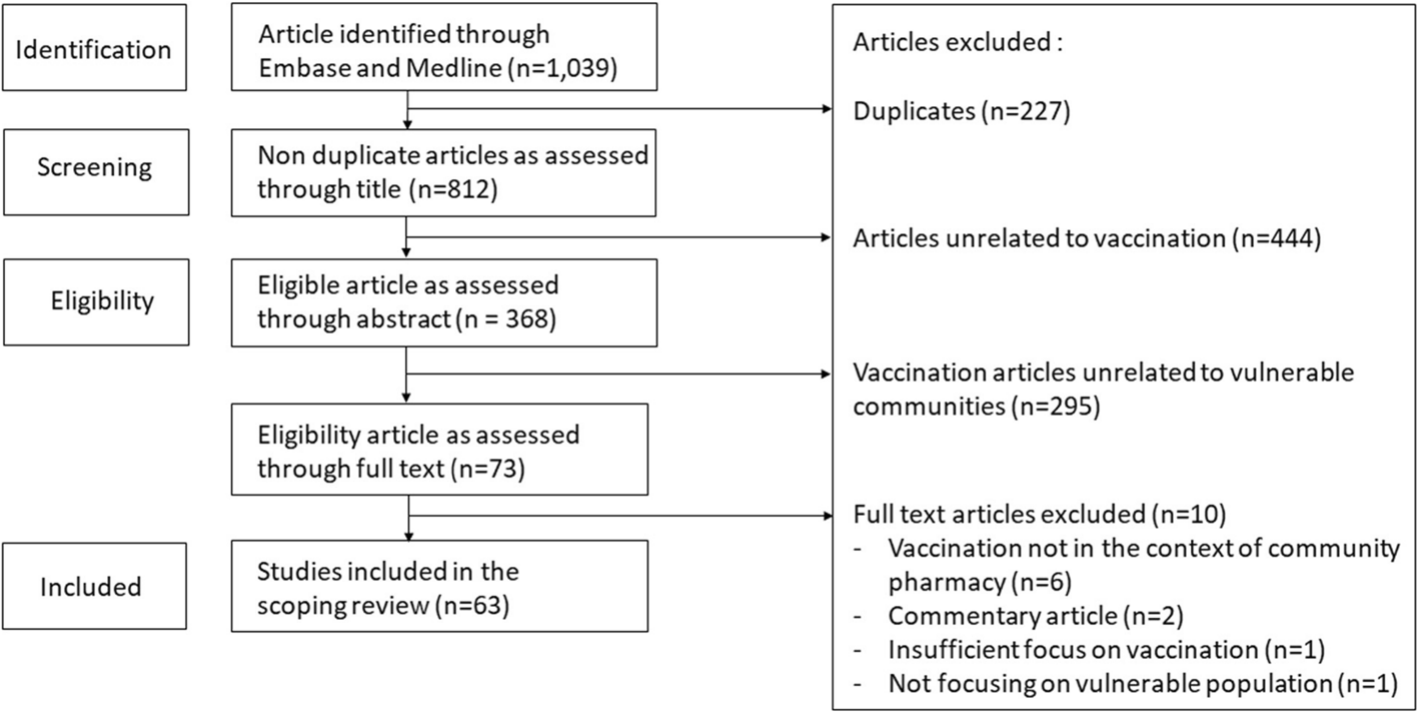
Study Selection Process Flowchart

After performing the initial search, 1,039 articles were identified (Fig. 1). We found 614 articles originating from the Embase database and 425 from the MEDLINE database. We removed 227 articles due to duplication within or across databases. The 812 remaining articles titles were screened, and 444 articles were removed because vaccination was not a central topic in the research. The remaining 368 articles were screened through their abstract and 295 articles were discarded because they did not address a population considered as vulnerable. The remaining 73 articles were fully read, and 10 articles were discarded since they occurred outside of a pharmacy setting, were not original research or focused insufficiently on vaccination or on a vulnerable population. The 63 included studies are presented in Table 1.

**Table 1:** Summary of Peer Review Articles Included in the Scoping Review

	**Authors**	**Year**	**Article type**	**Objective**	**Country**	**Vaccine**	**Population**	**Impact**	**Targeting/ intervention**	**Barriers/facilitators **
1	Daniel et al. [28] ^a,b^	2021	Mixed methods: implementation study (surveys and semi-structured interviews)	Feasibility and potential efficacy of free HPV vaccination in rural community pharmacies.	USA	Human papilloma virus	Adolescent from 10-18 years old on Medicaid	Increase of 158.8% in vaccine uptake and 24.4% in total revenue was observed. The intervention was received positively by local health providers. Pharmacies also saw increased prescription revenue through the initiative as an indirect benefit	Health communication campaign targeting parents through increased knowledge, awareness (poster, social media, leaflet, personalized letter) and culturally relevant material	Facilitator: collaboration with public health and pharmacy banner, culturally relevant material. The intervention addressed common barriers: cost, convenience and receiving a strong vaccine recommendation
2	Falope et al. [29] ^b^	2021	Qualitative: semi-structured interview	Explore the knowledge and perceptions of Florida pharmacists administering vaccine	USA	Influenza	Pregnant women	Participants were knowledgeable about the influenza vaccine and its indication in pregnancy. Most pharmacists displayed a positive attitude towards various aspects of vaccination (access, expertise, increased scope of practice and ease of practice). They expressed a less positive view towards education.	Not specified	Facilitators: more technicians, marketing, education, better incentives, vaccine coverage and rural setting of pharmacy, provider referral, more patient education Barriers: access to pregnant women, increased workload, pharmacy environment and rural setting of pharmacy, patient awareness
3	Fathima et al. [30] ^a,b^	2021	Quantitative: quasi-experimental: pre-post pilot study	Evaluate the effectiveness of a COPD management program including discussion on immunization and prompt for vaccination at the 3rd visit	Australia	Pneumo-coccal and influenza	COPD patients of 40-80 years old, taking > 5 medications or > 12 doses per day	At the end of the study, pneumococcal vaccination status significantly improved. Flu vaccination status did not significantly improve.	Proving a strong recommendation for vaccination, screening during the work and through a chronic condition program	Facilitator: intervention led by a consultant outside the pharmacy work chain
4	Gatwood et al. [31] ^a,b^	2021	Quantitative: quasi-experimental: randomized pre-post study	Evaluate the impact of a communication training program to improve pharmacist promotion of the pneumococcal vaccine among high-risk adults	USA	Pneumo-coccal	High risk adults	The training program does not statistically increase the self-efficacy of pharmacists but increased absolute percentages. Percentage of pneumococcal vaccination declined in all groups except in the full training group.	Assertiveness communication training program to pharmacists (online or online and in person), strong recommendation from healthcare professional	Barriers: wider vaccination promotion both in the community and within the store are necessary, time constraints require initiatives to improve workflow such as reminder systems to identify high risk patients
5	Guadamuz et al. [32] ^b^	2021	Quantitative: retrospective multiple cross-sectional series	Evaluate trends and disparities in access to pharmacies in 4 largest cities in the United States, New York City, Los Angeles, Houston, and Chicago, by neighborhood racial and ethnic composition from 2015 to 2020.	USA	No specific vaccine	Racially and ethnically segregated neighborhood	More independent pharmacies were found in diverse, Black and Latino than in White neighborhoods and were less likely to offer immunization. Unequal access to pharmacy services may worsen health disparities in low-income neighborhoods	Not specified	Facilitator: chain pharmacies offer more financial resources, training and accommodations which facilitate the provision of medication and immunization services for pharmacists
6	Koskan et al. [33] ^b^	2021	Quantitative: cross-sectional survey	Assess attitude and behaviors of pharmacists and pharmacy interns on HPV vaccination	USA	Human papilloma virus	Children and adolescent	Many pharmacists seldom provide HPV vaccination but show positive attitude towards this vaccine. Pharmacists’ intention to vaccinate correlates with their behavior.	Not specified	Barriers: obtaining vaccination consent from parents, parent’s stigma against HPV and prescription requirement Facilitator: education of the parent and booster reminder system
7	Liao et al. [34] ^b^	2021	Quantitative: retrospective multiple cross-sectionnal series	Assess the trends in location where influenza vaccine was received between community pharmacy, physician’s office/clinics and other places between 2008 to 2015	USA	Influenza	Adults > 65 years old on Medicare	Influenza has slightly increased in the older population. Vaccination in pharmacy gradually increased in comparison to doctor’s office or clinic	Not specified	Facilitator: pharmacies are not competing with physician office and clinic for providing vaccination services but instead complement them by adding additional access points
8	Lu et al. [24] ^b^	2021	Quantitative: cross-sectional multivariable logistic regression	Analysis of the characteristic of patients getting their vaccination in medical and non-medical sites	USA	Influenza	Chronic conditions, age, race/ethnicity	Individuals with higher education than a high school degree, annual household income more than $50,000, those without a doctor’s visit since July or those having a doctor’s visit since July but no recommendation for influenza vaccination were more likely to get vaccinated in pharmacy. Non-Hispanic Blacks, Hispanics, multiple races, those never married, unemployed adults and those living in Western region of USA were more likely to receive their vaccines in medical settings.	Not specified	Barrier: prescription requirement in some states
9	Neuner et al. [35] ^b^	2021	Quantitative: retrospective cohort study	Determine whether pharmacy access is associated with influenza vaccination in subjects recently diagnosed with breast cancer, and whether this association differs by additional risk factors for influenza complications	USA	Influenza	Patients > 65 years old with nonmetastatic breast cancer on Medicare	Black, Hispanic, Medicaid beneficiaries, patients diagnosed in autumn and patients living in low-access census tracts were less likely to receive a vaccine. Patients with higher comorbidity and lower cancer stage were associated with higher vaccination.	Not specified	Barrier: access to a pharmacy does not reduce disparities in vaccination according to race, ethnicity and census tract
10	Nuffer et al. [36] ^a,b^	2021	Quantitative: retrospective cohort study	Three years follow up of a 6-month chronic disease intervention was performed and examined various outcomes including vaccination status.	USA	Pneumo-coccal, influenza	Diabetic and cardiac patients of rural communities	Enrolled patients showed higher pneumococcal than influenza increase in vaccination status. More patients remained unvaccinated for influenza after the program. 20% of patients underwent the 6 visits of the program. Largest decline was between visit 1 and 2, suggesting that the structure of the education or the nature of the consult was not what participants expected.	Promotion through posters, word-of-mouth, leaflet, personalized phone call, conversation initiated by staff, provision of strong recommendation for vaccine, staff training, generating a list of eligible patients, screening through an existing program	Facilitator: providing a strong recommendation for vaccination Barrier: missed opportunities, difficulty to reach prospective patients
11	Tyler et al. [37] ^a,b^	2021	Quantitative: retrospective cohort study	Analyze the impact of a pharmacist phone call and cost on the completion of the 2nd dose administration.	USA	Herpes zoster	Patients having received 1 out of 2 doses of shingles vaccine	Patients receiving an intervention from the pharmacist were more likely to receive the 2nd dose. The cost of the vaccine did not affect the likelihood to receive the 2nd dose	Personalized phone call intervention (reminder and clinical information if requested)	Facilitator: implementation of a dose tracking history and call list was possible in different pharmacy systems. It could be made possible for other vaccines
12	Beal et al. [38] ^b^	2020	Systematic review	Impact of pharmacist on realized accessibility, financial accessibility and vaccine accessibility	USA	Influenza, pneumococcal, herpes zoster	Adults > 65 years old	Majority of studies centered around realized accessibility, one on financial accessibility and eleven on vaccine availability. Only 20% of studies included pharmacists as documenters. The role of immunizer is preferred for cost-saving impact for pharmacies and insurance companies.	Not specified	Barriers: lack of knowledge, lack of opportunity for vaccination, financial cost and vaccine accessibility
13	Coley et al. [39] ^a,b^	2020	Quantitative: quasi-experimental pre-post study	Demonstrate the impact of a notification and motivational interviewing processes at a regional supermarket chain pharmacy to increase the number of vaccines	USA	Influenza, pertussis, pneumococcal, herpes zoster	High risk patients eligible according to age and prescription information	A 33% increase in vaccination was observed. All vaccines but shingles increased (influenza +45%, pertussis +31%, pneumococcal +7%, shingles -5%). An increase in patient’s readiness was observed with motivational interview	Generating a list of eligible patients, printing a note on medication bag, motivational interview training to staff and face-to-face or telephone motivational interview	Facilitator: seasonal approach to vaccination helped managing the workload, support from pharmacy chain, training provided to all pharmacy staff Barrier: limited human resources, complex eligibility criteria
14	Deslandes et al. [40] ^b^	2020	Quantitative: longitudinal cohort study	Change in community pharmacy delivered flu vaccines since the NHS flu vaccination program from 2012-2018	UK	Influenza	Adults > 65 years old and at-risk adults < 65 years old	A 20-fold increase in vaccination in community pharmacy was observed between 2012-13 and 2017-18 A strong positive correlation was observed between increasing community pharmacy vaccination and total number of vaccinations	Providing convenient modalities for walk-in	Facilitator: increase vaccination in community pharmacy did not reduce the number of vaccines provided in general practitioner’s office, convenience (walk-in)
15	Frederick et al. [41] ^a,b^	2020	Mixed methods: implementation study (surveys and semi-structured interviews)	Implementing a clinical decision support within the pharmacy software system alerting pharmacist of eligible patients for a 2nd dose	USA	Herpes zoster	Recipients of 1st dose	Most pharmacists agreed or strongly agreed that the intervention is acceptable, appropriate and feasible in a community pharmacy setting.	Screening during the workflow through an eligibility nudge	Facilitator: integration of the system in the workflow, patient trust, team’s willingness to participate and engagement
16	Gauld et al. [42] ^a,b^	2020	Qualitative: semi-structured interviews	To explore the effect of funding maternal vaccinations through community pharmacies on accessibility, uptake, awareness and the views of health professionals and patients on the service, barriers and enablers to uptake.	New Zealand	Pertussis, influenza	Maori/Pacific and non-Maori pregnant women	Most views of vaccination extension in pharmacy were positive. Vaccination in pharmacy will increase awareness. Some pharmacists report high maternal vaccination uptake.	Pharmacists were offered a training, patients were reached through promotion endeavors (poster, social media, leaflet) and verbal conversation.	Facilitators: convenience, access, proactivity, interest, qualified staff, communication with other health providers, promotion Barriers: too busy lack of training, insufficient staff, interest, reaching prospective patients, vaccine distribution
17	Krueger et al. [43] ^b^	2020	Quantitative: randomized controlled trial	Impact of a science-based communication on attitude towards pneumococcal vaccination in a community pharmacy	USA	Pneumo-coccal	Non-White adults	Community/family duty and combination messages showed significant influence on attitude for non-Whites. Combining duty to family and friends, fatality and safety significantly improved the intention to ask a medical professional about the vaccine.	Culturally adapted promotional campaign, provision of strong recommendation for vaccination	Facilitator: culturally adapted communication campaign, provision of strong recommendation for vaccination Barrier: non-White patients are less likely to follow health and medical recommendations which may decrease the odds of clinicians communicating the types of messages in this study.
18	Page et al. [44] ^a,b^	2020	Quantitative: quasi-experimental pre-post study	Evaluate the impact of a pharmacist education and intervention vaccine rates. Assess patient’s awareness and barriers to receiving the polysaccharide pneumococcal vaccine.	USA	Pneumococcal	Diabetic patients of 19-65 years old	Pharmacist intervention significantly improved the vaccination status (+18%). Intervention rate improved the pneumococcal vaccination status of diabetic patients from 28.6 to 31.8%	Generating a list of diabetic patients, a note was added in eligible profiles, education and recommendation of vaccination was provided at the next encounter	Barrier: desire to discuss vaccination with their primary physician, time constraints, unawareness of vaccine need
19	Singh et al. [45] ^a,b^	2020	Quantitative: retrospective cohort study	Evaluate the effect of a free flu vaccine voucher in pharmacy during 2015-2016 and 2016-2017 on hospitalizations, ambulatory care visits, death and costs.	USA	Influenza	Uninsured adults	1 million vouchers were distributed (600 000 in 2015-2016 and 400 000 in 2016-2017) with respective redemption rates of 52% and 87%. The program potentially avoided 13 347 cases, 4622 ambulatory care visits, 168 hospitalizations and 8 deaths during the 2^nd^ year. It generated health care savings of 19.5 million $ in total societal costs.	Providing free vaccine vouchers	Facilitator: accessibility, financial aid for vaccine, tailoring distribution to improve redeeming rates (distribution through community organizations), cost-saving initiative
20	Spinks et al. [19] ^b^	2020	Systematic review	Review of the impact of permitting pharmacists to vaccinate regular and at-risk population.	USA, Canada, UK	Influenza	Adults > 65 years old	Differences in population vaccination rate for > 65 years old associated to pharmacists varied from 0.4-11% There was evidence of substitution by pharmacists, but the effect was small.	Not specified	Facilitator: pharmacists with the most autonomy demonstrated largest increase. Vaccination by pharmacists appears cost-saving
21	Teeter et al. [46] ^b^	2020	Mixed methods: implement-tation study (survey and semi-structured interviews)	Identify the barriers and facilitators to community pharmacies’ provision of HPV vaccines, select and implement a physician-pharmacist collaboration model	USA	Human papilloma virus	Adolescents of 11-17 years old	Identification of 3 collaborative models: ‘’shared responsibility model’’ (1st dose given by doctor and 2nd by pharmacist), ‘’pharmacy-based state management protocol model’’ (strong recommendation by physician to receive 2 doses at pharmacy)’’ and ’’in source model’’ (physician invites pharmacist to give vaccine clinic at their office).	Partnership with other providers, strong recommendation from a healthcare professional	Barrier: requirement of patient specific prescription or disease state management protocol complexify the implementation for providing HPV in pharmacy
22	Zahnd et al. [47] ^b^	2020	Quantitative: geospatial analysis	To assess if geographical access to pharmacies amongst adolescents and adults in South Carolina according to rurality and access to primary provider	USA	Human papilloma virus	Adolescents in rural areas	Areas with higher access cluster around metropolitan area. Spatial access was higher in metropolitan areas than micropolitan areas. Micropolitan and small towns have similar access. Health provider shortage areas are also linked with low spatial access to pharmacies. In micropolitan area, there is no difference in access to pharmacy in health provider shortage area or in non-health provider shortage areas.	Not specified	Facilitator: pharmacies are more available even in health provider shortage area. Capacity of pharmacy to store vaccines, provide insurance coverage, state laws and policies are important factors to consider in the provision of vaccines. Barrier: not all states allow pharmacist to prescribe and administer vaccination.
23	Ariyo et al. [48] ^a,b^	2019	Quantitative: quasi-experimental pre-post study	Characterize the medication therapy problems and vaccines recommended /administered at appointment-based medication synchronization visits in community pharmacies	USA	Hepatitis A, hepatitis B, herpes zoster, influenza, measle-mumps -rubella, meningo-coccal, pneumo-coccal, diphtheria-pertussis-tetanus	Older adults > 65 years old and 18-64 years old with more than 3 chronic medications	184 patients participated. 633 vaccines were recommended during the initial visit. 51 vaccines were administered. 17 minutes was reported for the initial visit. In person consultations were associated with more vaccine administrated.	Generating a list of eligible patients, screening during workflow or during another program	Barrier: new services take time for the staff to become comfortable and learn to incorporate in the workflow, timing of recommendation was too early for some patients to receive the influenza vaccine in September, preference to receive vaccine at the physician’s office or unsure if they received
24	Calo et al. [49] ^a,b^	2019	Mixed methods: implem-entation study	Process evaluation of HPV vaccination in pharmacies of 5 states (Oregon, Iowa, Kentucky, Michigan and North Carolina) and documenting real-world pharmacy settings.	USA	Human papilloma virus	Adolescent and young adults	Sites showed low or no service penetration. 13 doses were given to adolescents and 3 to adults. No vaccines were given in Oregon. Key barriers were linked to service penetration, appropriateness, feasibility, adoption and sustainability barriers.	Posters in pharmacy, advertising, personalized letter, collaboration with physicians	Facilitator: acceptability of service and waiting times by parents, convenience, satisfactory privacy and confidentiality Barriers: resistance by some pharmacy staff demonstrated resistance due to engagement, staffing, workflow integration, funding delays, third-party reimbursement issues, physician collaboration reluctance and low patient awareness
25	Doucette et al. [50]^a,b^	2019	Quantitative: implementation study	Feasibility of a coordinated model of HPV vaccination where clinic provides first doses and pharmacy provide subsequent doses.	USA	Human papilloma virus	Adolescent and young female adults (mean age 13 years old)	51 patients were referred to the pharmacy. 28 declined. During the study, 25 vaccines were given to 23 patients (12 months period). All patients completed their HPV series.	E-prescription facilitated the prescription order for 2nd and 3rd dose. Information flyer and text messages were implemented to remind patients of the appointment	Facilitators: patient appreciated receiving information from pharmacists, combination of 2 voices to provide a stronger recommendation to address hesitancy Barriers: few interested patients, workflow integration, lack of staff time and some language barrier, lack of access to an electronic health record for pharmacists
26	Reidenbach et al. [51] ^b^	2019	Quantitative: cross-sectional needs assessment study	To describe the preconception care needs among female patients of a community pharmacy	USA	Influenza, hepatitis B, diphtheria-pertussis-tetanus, measle-mumps-rubella	Women of childbearing age	78.8% of women were missing documentation on one or more recommended vaccines	Screening for vaccination status amongst other preconception care. A standardized letter was sent to women with incomplete vaccine record or missing vaccine encouraging vaccination.	Facilitator: providing care, counseling or referral Barrier: lack of patient awareness on vaccine need, vaccine may be done at other pharmacies leading to discrepancies
27	T Bach et al. [52] ^b^	2019	Quantitative: cross-sectional study	Evaluation of a convenient sample of 11 community pharmacies’ screening form for pharmacists to make proactive recommendations	USA	Influenza, pneumo-coccal, herpes zoster, human papilloma virus, meningo-coccal, diphtheria-pertussis-tetanus	Adults older than 65 years old, chronic conditions (heart, liver, kidney, lung conditions, diabetes), pregnant women, adolescents	8669 vaccine screening forms were analyzed in 1 year. Influenza vaccine was the most popular administrated vaccine (75%). Patients have on average 1 vaccine recommended besides influenza vaccine. 10 and 35% of patients were indicated for herpes zoster or herpes zoster+ pneumococcal. Although 10/11 pharmacy ask about pregnancy, 22% of women received the Tdap vaccine during their consultations.	Not specified	Facilitator: screening tools help identifying missed opportunities Barrier: increase paperwork and workflow burden for patients and pharmacists
28	Waite et al. [23] ^b^	2019	Quantitative: cross-sectional study	Assess the characteristics and predictors of patients receiving vaccination at the pharmacy compared to physician’s office	Canada	Influenza	Age, income, race, chronic condition, contact with pharmacy, history of hospital admission	Living in a nonurban area or higher income neighborhoods, not identifying as immigrant, not having diabetes or hypertension and receiving a pharmacist service the same day were predictors of vaccination in pharmacy. For > 65 years old, having a hospital admission during the year correlated with pharmacy vaccination whereas higher annual medication cost correlated with physician’s office.	Word-of-mouth, pharmacy specific advertising	Facilitator: proportion of patients vaccinating in pharmacies is increasing due to availability of the service through public health
29	Colorafi et al. [53] ^b^	2018	Qualitative study: semi-structured interviews	Descriptive analysis of pharmacy barriers to pneumonia vaccination in 2 rural counties of Washington	USA	Pneumococcal	Rural counties	60% of pharmacists vaccinated against pneumonia. Some pharmacies chose not to vaccinate not to disrupt the existing collaborative relationship with physicians. Some pharmacies required prescriptions from a physician to administer. Patients acted like consumers to find the best price.	Convenient methods for patients such as walk-in were made available.	Facilitator: walk-in modalities Barriers: competing priorities during patient visits, failure to assume responsibility to educate and vaccinate, challenges in determining vaccination status, knowledge gaps, complexity of recommendation, lack of vaccine availability. Pricing variability affects perception of affordability and need for prescription affects acceptability
30	Klassing et al. [54] ^a,b^	2018	Quantitative: randomized controlled trial	To determine if pharmacy-initiated interventions improved the rate of influenza and pneumococcal vaccinations	USA	Influenza, pneumo-coccal	Adult patients with asthma and COPD	Control participants resulted in significantly higher influenza uptake than letter or phone call group. Letter group resulted in higher pneumococcal uptake although not significant. Sub-analysis of patients under 65 years old resulted in significantly higher influenza rates in the letter group compared to the phone call group.	Receiving a standardized letter or receiving a personal phone call recommending influenza and pneumococcal vaccination	Facilitators: marketing initiatives improves awareness and vaccination rates Barrier: difficulty to reach patients through phone or mail, many patients expressed the desire to discuss vaccination with physician
31	O’Brien et al. [55] ^a,b^	2018	Quantitative: implement-tation study	Describe the methods and perspectives on the first outpatient pharmacy to provide influenza vaccination to military personnel	USA	Influenza	Army soldiers, health care professional	Implementation was simple once the preparation was completed. In 2016-2017, 238 people received the vaccine. In 2017-2018, 761 people received the vaccine (about 2/3 of beneficiaries /employees – 1/3 soldiers)	Promotional posters in pharmacy, social media advertising, promotion to other health providers, leaflets, collaboration between provider, staff training	Facilitator: partnership with public health instances helped getting a standing order for a pharmacist to vaccinate
32	Patel et al. [56]^b^	2018	Quantitative: quasi-experimental longitudinal time series, logistic regression	Odds of being immunized after exposure to pharmacy service compared to before the service	USA	Influenza, pneumococcal	High-risk population including people aged > 65 years old	Exposure to pharmacy immunization services increases the likelihood of pneumococcal and influenza vaccination by 2.5% and 1.5%.	Not specified	-
33	Shah et al. [57] ^b^	2018	Quantitative: geospatial analysis	Comparison of spatial dispersion of pharmacies and physician’s office to assess adequate access to vaccine in Texas.	USA	Human papilloma virus	Adolescent	High per capita physicians were located near other high per capita physician census tracts (cluster). Pharmacists are more geographically dispersed than physician’s offices. Adding them as vaccine providers in area of inadequate coverage improves vaccine availability (33-55% coverage). Urban areas saw higher improvement than rural area (35% vs 18%)	Not specified	Facilitator: pharmacies are encouraged to be geographically dispersed to avoid competition. Economies of scope by providing different vaccine services can be achieved in pharmacies. Diversification may be a business strategy
34	Wick et al. [58] ^a,b^	2018	Quantitative: quasi-experimental pre-post study	Define the perception and awareness on HPV vaccination in pharmacies. Describe parental intentions and rationale in providing HPV vaccination to their child. Assess the impact of a pharmacist-led education group session.	USA	Human papilloma virus	Parents of children < 9 years old	The intention to vaccinate increased by 9% (35-44%) and participants against vaccinating decreased by 11% (23 to 12%) The intervention Increased awareness of availability of HPV vaccine in pharmacy from 32-100%	Pharmacy led education group sessions	Barrier: most participants had made their decision before the child’s birth. Early intervention when the decision is made may be efficient to reverse vaccine hesitation.
35	Bedwick et al. [59] ^a,b^	2017	Quantitative: quasi-experimental pre-post study and survey	Impact of an automated phone message from the pharmacy owner recommending the vaccine on vaccination rates and patient satisfaction	USA	Herpes zoster	Adults > 60 years old	25 patients received the vaccine during the study period. Receiving the phone call was the most cited reason followed by doctor’s recommendation to get vaccinated. 16/18 receiving the phone call reported that the phone call influenced their decision. Patients showed high satisfaction with the method.	Personalized phone call providing a strong recommendation for vaccination	Facilitator: cost-effective intervention Barriers: workflow disturbances on the day of the messages due to an increase in the volumes of calls, necessity for all pharmacists to be informed of the content of the phone message, complexity to remove patients that received the message from the list, difficulty to have an updated list of patient’s phones
36	Di Pietro Mager et al. [60] ^a,b^	2017	Quantitative: implementation study	To demonstrate the ability of a statewide network of community pharmacists to provide preconception care services with the use of targeted medication reviews	USA	Measle-mumps-rubella, hepatitis B	Women of childbearing age (15-45 years old)	1149 pharmacists from 818 pharmacies participated. 3844 patients with immunization opportunities were identified. 1411 (37%) target medication review were performed. 971 (69%) of those received immunization services.	Pharmacist training, generating a list of eligible patients, screening during clinical program	Facilitators: minimal training and support are required Barriers: targeting could be done more specifically to women wishing to conceive
37	Fava et al. [61] ^a,b^	2017	Narrative review	Review of the literature on barriers and initiatives in HPV vaccination	USA	Human papilloma virus	Adolescent	Literature on pharmacy-based programs is scarce compared to health systems and public health driven models. Only 1 pharmacy-based HPV program targeting underinsured college students was found. *Vaccine for Children* program allows free vaccination for American Indian but only 100 pharmacies are providers.	Text-based reminder, phone reminder	Barriers: cost, lack of access, misinformation regarding vaccines, reaching adolescent, social philosophical religious stigma among parents as a barrier to effective provider information and recommendation, staff training, vaccine access, training and complexity with *Vaccine for Children* program in pharmacies.
38	Inguva et al. [26] ^b^	2017	Quantitative: cross-sectional study, logistic regression	Assess the characteristics of patients receiving vaccines in a pharmacy setting across 8 US states and Puerto Rico	USA	Influenza	Age, race and state of origin in the USA	Doctor’s office is the most prevalent site of vaccination (37.5%) followed by community pharmacy (23.3%). Older adults, multiracial participants, Hispanic respondents and residents of states that allowed vaccination before 1999 were more likely to use pharmacy services. Poor health, having high risk conditions, Black and White responders are associated higher doctor’s office vaccination.	Not specified	Facilitator: physician’s order is no longer necessary for Medicare and Medicaid patients
39	Jimenez-Quinones [62] ^a,b^	2017	Quantitative: quasi-experimental pre-post study	To observe whether local vaccination rates are improved by a patient and physician education program on (HPV) in a community pharmacy of Puerto Rico.	USA (Puerto Rico)	Human papilloma virus	Adults in low socio-economic area	Out of the 200 candidate patients, 79 were reached. Only 7 patients received the educational session. 4/79 received the vaccine (1 had received the educational program).	Listing eligible patients, phone call to educate and counsel patients, invitation to an educational groups program	Facilitator: the pharmacy system was efficient at identifying the candidates for HPV, collaborative agreements helped access to HPV vaccines. Barrier: refusal to participate in group program was associated to lack of time.
40	Kulczycki et al. [63] ^b^	2017	Qualitative study: semi-structured interviews	Assess the knowledge, practice patterns of community pharmacists, challenges to offering pneumococcal vaccines and determine opportunities for expanding community pharmacy-based vaccination services in Alabama.	USA	Pneumo-coccal	At-risk adult 19-64 years old (chronic conditions) and older adults > 65 years old	Several knowledge gaps were identified in the target population pneumococcal recommendations. Most pharmacists did not fully utilize the available data to target or promote vaccination. The vaccine is rarely recommended outside the flu season.	Screening through during influenza vaccination services	Barriers: advocacy, public misperceptions, limited collaboration with physicians, resource constraints, improving the record-keeping system, patient-pharmacist trust Facilitators: growing acceptance of pharmacy-based immunization, business case for pneumococcal, interest for continuing education
41	Pattin [64] ^b^	2017	Narrative review	Review of pharmacy technician’s role in reducing immunization disparities.	USA	Influenza, pneumo-coccal, herpes zoster	Racial and ethnic disparities	Overall vaccination is low and fails to meet goals. Technicians play a role in improving vaccination.	Pharmacy technician initiation of conversation on vaccine	Facilitator: trust in provider, opinion leader Barrier: lack of knowledge among health care provider and consumers on the benefits of vaccination, staying current, lack of knowledge of immunization disparities, cultural and language difference provides distrust, distrust in physicians, personal beliefs against vaccines, lack of insurance coverage, difficulty to assess patient’s eligibility
42	Anderson et al. [14] ^b^	2016	Quantitative: cross-sectional study	Assess the characteristics of patients receiving vaccination at the pharmacy compared to other vaccination site.	UK	Influenza	Age, education, health care workers	1741 questionnaires were obtained from 55 pharmacies. 19% of vaccinated patients in pharmacies. Older adults, health care workers and more educated patients were more likely to be vaccinated in pharmacies than at other sites.	Not specified	-
43	Hohmeier et al. [65] ^a,b^	2016	Mixed methods: implementation study (surveys and semi-structured interviews)	Describe and report the impact of a multimodal series of pharmacy led educational intervention targeting eligible patients in community pharmacy	USA	Human papilloma virus	Adolescents and adults 9-26 years old	No patients received the vaccine during the control period and 10 vaccines were dispensed during the intervention period (9 1st dose, 1 2nd dose).	Education during dispensing, poster, flyers, provider education, customized prescription pads	Facilitator: pharmacist recommendation to improve awareness, physician’s recommendation, convenience attracted patients Barrier: lack of insurance coverage
44	Eid et al. [66] ^b^	2015	Narrative review	To review the impact of pharmacist intervention on herpes zoster vaccination rates	USA	Herpes zoster	Adults > 60 years old	2 studies on active promotion by pharmacists were found. Bryan et al. showed significant rise (12.1% vs 1.5%) in vaccination through training of staff with personal selling training, personal letter, pharmacy technician initiation of conversation and passive promotion. Wang et al. showed significant increase in vaccination (1.2% vs 0.37%) with the use of promotional material sent to patients. A personalized letter was the most effective for of pharmacist intervention.	Staff training, face-to-face interaction, education, promotional material (newspaper, flyers, personalized letters)	Facilitator: staff training, working with interns and technicians to initiate the conversation, marketing, recruiting patients outside of the pharmacy work chain
45	Liu et al. [67] ^b^	2014	Quantitative: cross-sectional study	Estimating the rate of vaccination of adults over 60 years old in community pharmacies	Canada	Herpes zoster	Adults > 60 years old	Dispensing rates increased sharply from 2009-2013. 8.4% of Alberta residents > 60 years old received the vaccine as of 2013 in pharmacy. Most vaccines were dispensed to urban residents (87%), adults 60-69 years old (42.5%) and to women (9.5%)	Not specified	Barrier: coverage of vaccine remains a barrier to accessibility
46	Navarrete et al. [68] ^a,b^	2014	Quantitative: implementation study	Needs assessment and implementation of an HPV vaccine program at the pharmacy located in the university clinic	USA	Human papilloma virus	Underinsured university students (17-35 years old)	72% of students did not understand how HPV is transmitted. 89 patients (79.8%) received their 2nd doses and 48.3% completed the vaccine series. 46 patients did not complete the vaccination series due to follow-up loss or other reasons.	Promotion and marketing campaign, financial aid for vaccine coverage, vaccination, healthcare provider references	Facilitator: provision of vaccine via a physician signed protocol, references by other clinics and financial aid program Barriers: inadequate provider recommendation, lack of time, reimbursement, infrequent reminders / recall systems, parental hesitancy, discomfort talking about sexual health, lack of health care access
47	Teeter et al. [69] ^a,b^	2014	Quantitative: cross-sectional study	Document patient characteristics, awareness and knowledge on herpes zoster vaccine and reasons for not getting the vaccine. Assess the impact of an education program on interest in obtaining the vaccine.	USA	Herpes zoster	Adults > 60 years old	681 patients participated in conversations with pharmacy students. Most participants (73%) were interested in talking to a health provider after the education. People who did not have time to get the vaccine were the most interested in speaking with a pharmacist (91.5%)	Education program on herpes zoster vaccine provided by pharmacy student, strong recommendation from healthcare provider	Facilitator: convenience, suggestion to discuss vaccine with pharmacists or physicians Barriers: lack of time/forgot or did not know it was needed, cost of vaccine, lack of recommendation from physicians
48	Bryan et al. [70] ^a,b^	2013	Quantitative: quasi-experimental prospective comparison study	Comparison of promotional techniques (personal selling vs and personalized letter targeted to eligible patients).	USA	Herpes zoster	Adults > 60 years old	Significantly more patients made commitments to receive the vaccine with active promotion (12% vs 1.5%). Patients receiving a personalized letter made more commitments than patients receiving a phone call. Active promotion significantly improved patient’s attitude towards receiving the vaccine and reduced the average time spent with patients. Personal selling, friends/family, physician were more frequent reasons to get the vaccine than brochure and poster.	Personal selling, providing a strong recommendation for vaccination, personalized letter targeting, listing eligible patients, poster, leaflets	Facilitator: support from technicians, influence from family and friends, availability of the vaccine, strong recommendation from providers, awareness for vaccine Barriers: desire to discuss vaccination with physician, time constraints, staff support, legal liability, adequate space, reimbursement, lack of training, lack of perceived knowledge, poor upper management support
49	Hess [71] ^a,b^	2013	Quantitative: randomized controlled trial	Measure the impact of an automated outbound telephone messaging system on herpes zoster vaccinations	USA	Herpes zoster	Adults > 60 years old	Telephone significantly increased the rate of vaccination from 46 (0.72%) to 146 (2.6%) vaccines administered between the intervention and control group.	Automated telephone messaging system, sent to a list of eligible patients	Facilitator: novelty of the vaccine, trusted source of the message, the vaccine was not back ordered in the intervention, champions could influence the rates in some locations Barrier: lack of patient awareness
50	Wang et al. [72]^a,b^	2013	Quantitative: quasi-experimental pre-post study	To evaluate the effectiveness of community pharmacy–based interventions in increasing vaccination rates for the herpes zoster vaccine	USA	Herpes zoster	Adults > 50 years old	Vaccination rates significantly increased from 59 (0.37% of eligible patients) to 169 patients (1.20% of eligible patients). More patients reported being educated and influenced by the pharmacy-driven intervention. Flyer and newspaper were significantly cited as more effective interventions	Multisite promotional intervention: newspaper press release, advertisement flyer on all prescriptions and a personalized letter mailed to eligible patients	Facilitator: comfort with pharmacist administration of vaccine, collaborative agreements facilitated the obtaining of prescriptions Barrier: vaccination statistics decreased after intervention. Reinforcement may be necessary. Lacking collaborative agreement made vaccination more complicated
51	Murphy et al. [73] ^b^	2012	Quantitative: cross-sectional study	Assess the extent of Walgreen pharmacies provision of vaccines in MUA	USA	Influenza	Medically underserved community (MUA)	1.75 million of influenza vaccines were administered by Walgreens in MUA. Mississippi and New Mexico had the highest percentage of MUA and pharmacies provided 68.6% and 54% of all vaccines.	Not specified	Facilitator: long opening hours of pharmacies and convenience
52	De Bruyn et al. [74] ^a^	2011	Quantitative: cross-sectional study	Comparison of pharmacy delivered vaccines 2010-2011 to the previous year (2004-2010)	Belgium	Influenza	Adults > 50, years old, chronic condition (cardiac, pulmonary, immune-compromised, diabetic, kidney patients), institu-tionalized patients, healthcare professionals	Vaccination rates increase with age. The 95 years old + age bracket appears less vaccinated. Diabetic patients showed similar variations in vaccination rates in comparison to non-diabetic patients.	Not specified	Facilitator: H1N1 was a strong incentive for patients to get vaccinated, vaccine uptake appears linked to media attention to vaccination. Barrier: requirement for prescription and mention of the patient’s eligibility by the doctor
53	Durham et al. [75] ^a^	2011	Quantitative: quasi-experimental retrospective cross-comparison	Comparison between PCP (primary care providers) and PTC (pharmacist-run travel clinic) in provision of travel medication and vaccines. Comparison of adequate prescription, missed opportunities, inadequate prescription and compliance to recommendation	USA	Travel vaccines	Travelers	513 patients were seen (172 by PCP and 341 by PTC). PTC patients were ordered significantly more vaccines per patients when indicated (2.77 vs 2.31) and were significantly more likely to receive them (2.38 vs 1.95). PCP recommended significantly more vaccines not consistent with guidelines per patient. Activities planned and purpose of travel were more documented in PTC.	Not specified	Facilitator: providers with training allows for better recommendations, the intervention was well accepted
54	Skiles et al. [76] ^b^	2011	Quantitative: cross-sectional study	Assess the attitude of state pharmacy association delegates towards adolescent immunizations	USA	Human papilloma virus, diphtheria-pertussis-tetanus, influenza	Adolescent	24/50 states answered the survey. 14/24 allow adolescent vaccination. 4/14 require prescription. Minimal advertising of adolescent vaccination services exists. Most respondents believed in the importance of adolescent vaccination. 67% agreed that HPV vaccination recommendations were controversial. Knowledge of the minor consent laws was limited.	Not specified	Barriers: vaccine storage and handling, financing and collaboration with primary care provider, lack of patient awareness, vaccine hesitancy
55	Taitel et al. [77] ^a,b^	2011	Quantitative: quasi-experimental case control study	Impact of pharmacy education on pneumonia risk during influenza immunization, educate them and provide vaccination.	USA	Pneumo-coccal	Adults > 65 years old, patients with chronic conditions (pulmonary, cardiac, liver, immune-compromised, diabetic, asplenia patients)	2 million patients received an influenza vaccine, and 1.3 million patients were eligible to the pneumococcal vaccine (69% over 65 years old, 31% chronic conditions). Patients in the intervention group significantly received more vaccination than those in the benchmark group (4.9% vs 2.9%). Patients 60-70 years old had the highest rate of vaccination.	Screening during vaccination activities, strong recommendation from a provider, notification letter was then sent to the physician or given to the patient	Facilitator: providing concurrent vaccines Barriers: missed opportunities, limited setting, fear of adverse effects and lack of awareness
56	Usami et al. [78] ^a^	2009	Quantitative: randomized controlled cluster study	To determine if personal advocacy for influenza vaccination by community pharmacists affected the vaccination rate and number of patients with influenza	Japan	Influenza	Adults > 65 years old	1776 participants completed both surveys (881 in intervention, 895 in control group). Vaccination rate in the intervention group was significantly higher (82% vs 65%) and influenza infection was significantly lower (2/881 vs 11/895)	Pharmacists provided information on risk and benefits of influenza vaccination (leaflet, mailing, poster)	Facilitator: conversation yielded a more thorough understanding than leaflet or mailing, a short 5 min conversation did not disrupt the workflow
57	De Bruyn et al. [79] ^a^	2008	Quantitative: quasi-experimental pre-post study	Impact of a pop-up system in pharmacist software when renewing diabetic medication.	Belgium	Influenza	Diabetic patients over and under 65 years old	14% of pharmacies sent a total of 420 standardized notes to doctors. 207 patients purchased the vaccine. Vaccination of diabetic patients increased by 2% between 2006-2007 and 2007-2008. Young diabetic patient vaccination rate increased by 4.6% compared to older diabetic patients which remained stable.	Pop-up nudge, pharmacists are then asked to discuss vaccination, give a pamphlet, send standardized note to doctor and encourage patients to contact their doctor	Facilitator: pharmacists are well suited to target diabetic patients Barrier: delay in vaccine reception, more collaboration between pharmacists and physicians can improve vaccine related communication, awareness of patient to vaccines
58	Marrero et al. [80] ^a^	2006	Quantitative: randomized controlled trial	Evaluate the education need of older adults, design, implement and evaluate a vaccine education program in pharmacy	USA (Puerto Rico)	Influenza	Adults > 65 years old	3 months after the study, 68% of the experimental group was vaccinated after phase 3 (vs 32% of the control group). 1 year after the study 72% of the experimental group was vaccinated (vs 24% of the control group) The experimental group had higher knowledge on the vaccine at 3 and 12 months. Patients that assisted to the education program did not visit a doctor for respiratory reasons.	Discussion groups, distribution of pamphlets, providing a strong recommendation for vaccination, collaboration the public health to obtain vaccines and nurse to perform immunizations	Facilitator: patient showed sustained, strong provider recommendation satisfaction toward pharmacist services, collaboration with nurses and public health Barrier: difficulty to reach prospective patients, vaccine availability
59	Hind et al. [81]^a^	2004	Quantitative: implem-entation study	Describe the impact of a new model of administrating influenza vaccine through community pharmacy and its uptake.	UK	Influenza	At-risk patients < 65 years old (diabetes, cardiac, pulmonary disease, immuno-compromised or carer of an at-risk patient)	56 patients were vaccinated in 1 pharmacy. 55 thought that the injection went as well as in the past. 10 would not have been vaccinated if not offered by the pharmacists. 46 would have gone to their general practitioner. Patient showed high acceptance of the intervention.	Posters, leaflets, proving a strong recommendation for vaccination by the pharmacist, interprofessional collaboration, screening eligible patients through the workflow	Facilitator: convenience in location, time, professionalism and adequate privacy, support from physicians, collective order, vaccine coverage
60	Barbero et al. [82]^a^	2003	Quantitative: quasi-experimental comparison study	Evaluate the performance and the number of travel health consultation to high-risk destinations in community pharmacy.	Spain	Travelling vaccines	Travelers to high-risk destinations	825 vaccines were recommended: 73% by pharmacists, 27% by doctors. 45% of patients received the vaccines recommended. More patients obeyed to the recommendation in trips to India and North Africa vs Caribbeans. 6.3% of patients received both correct vaccines and medications.	Specialized training program given to pharmacists, promotion through travelling clinics and health delegations, leaflets, collaboration with physicians for prescriptions, strong recommendation for vaccines was provided	Facilitator: accessibility, collaboration with health delegations, collaboration with physicians Barriers: bureaucratic barriers to obtain prescriptions, lack of patient awareness for risk
61	Ndiaye et al. [83] ^a^	2003	Mixed methods: cross-sectional surveys and semi-structured interviews	Perception of community and individual factors that influenced parents’ utilization of pharmacies through PIP (Pharmacy Immunization Program).	USA	Children vaccines	Medically underserved adults and children	6 interviews and 398 (96 PIP users, 302 non-PIP users) surveys were compiled. 8 main hypothetically influential factors were identified. 5/8 were significantly associated with pharmacy immunization: reliance/trust, timing, income, access/location, contacts/connections.	PIP was advertised through newspaper, TV, posters and handouts distributed in schools, schools, daycare, stores and pharmacies	Facilitator: providing immunization outside of parents’ work hour, accessibility without appointment, vaccines free of charge, convenience, trust, promotional collaboration
62	Grabenstein et al. [84] ^b^	2001	Quantitative: retrospective cohort study	Measure of the association between vaccination status and the availability of pharmacists as immunizer	USA	Influenza	Adults > 65 years old, chronic conditions (pulmonary, cardiac patients, diabetes)	The increase in influenza vaccination rates amongst 65+ was not significantly in state where pharmacists administer vaccines. Influenza vaccination rates amongst chronic conditions increased nearly significantly in state where pharmacists administer vaccines.	Not specified	Facilitator: rights to administer vaccines, patient awareness of vaccine services offered in pharmacy Barrier: patients are more likely to return to traditional providers than non-traditional providers
63	Rosenbluth et al. [85] ^a^	2001	Quantitative: implementation study	To describe the Pharmacy Immunization Project, a pharmacy/county health department partnership model for immunizing infants and adults in rural areas, and to develop service procedures and disseminate lessons learned for adapting the model to different settings.	USA	Children vaccines	Children vaccination in rural areas	All participants were satisfied and would recommend it. 4% preferred not getting vaccines at the pharmacy because they preferred their physician, they need more information, or the pharmacy is too busy. 4 out of 5 pharmacies continued the service. 1 dropped due to lack of demand. There were no problems or complaints from health care providers in the region.	Pharmacy and country health department partnership, collaboration with nurses to provide vaccines yearlong through a standing order, promotions via posters, flyers, direct communication, TV, radio and newspaper adds, presentations made by pharmacists to the community	Facilitator: facilitated process for states that do not allow pharmacists to immunize, trust in pharmacists, accessible services on Friday and Saturday nights, coordination between clinics and pharmacies helped solving scheduling difficulties, collaboration by physicians, patient awareness, strong recommendation by provider

^a^Studies that are part of a structured vaccination program

^b^Studies in settings where pharmacists are allowed to vaccinate at the time of the study

Most of the articles obtained were current, as 44 articles were published after 2014 (69.8%) (Table 2). Studies become scarcer as the further we investigated back in time. Eleven articles date from 2010–2014 (18%), 3 articles from 2005–2009 (5%) and 5 articles were published before 2004 (8%). No article included in our scoping review was published before 2000. Most articles originated from North America (*n* = 53, 84%) and a few articles came from Europe (*n* = 6, 10%) and Oceania (*n* = 2, 3%). Articles from North America collected data almost exclusively in the United States (*n* = 50, 79%). Three articles were conducted in Canada (5%). In Europe, articles originating from the United Kingdom (*n* = 3, 5%), Belgium (*n* = 2, 3%) and Spain (*n* = 1, 2%) were reported. One article was published from Australia (2%) and one from New Zealand (2%).

**Table 2:** Included study characteristics

Study Characteristics	Frequency	% (*n*=63)
**Qualitative**		
Semi-structured interview	4	6.3%
**Quantitative**		
Cross-sectional study	16	25.3%
Cohort study	6	9.5%
Quasi-experimental: Pre-post design	9	14.3%
Quasi-experimental: Case–control study	1	1.6%
Quasi-experimental: Comparison study	3	4.8%
Randomized trial control	5	7.9%
Implementation study	6	9.5%
Geospatial analysis	2	3.2%
**Mixed Methods**		
Mixed method*s*	6	9.5%
**Review**		
Systematic review	2	3.2%
Narrative review	3	4.8%
**Study Location**		
North America	53	84.1%
Europe	6	9.5%
Oceania	2	3.2%
Other	2	3.2%
**Publication Date**		
2015 +	44	69.8%
2010–2014	11	17.5%
2005–2009	3	4.8%
2000–2004	5	7.9%
**Vaccines**		
Influenza	29	46.0%
HPV	14	22.2%
Pneumococcal	14	22.2%
Herpes Zoster	14	22.2%
Tetanus, diphteria, pertussis	6	9.5%
Travel vaccinations (meningitis, hepatitis, typhoid fever, yellow fever …)	2	3.2%
Other	9	14.3%

The studies showed a wide variety of study designs with a predominance for quantitative frameworks (*n *= 48, 76%). A smaller portion of studies used qualitative design (*n* = 4, 6%), mixed-methods design (*n *= 6, 10%) and literature reviews methodologies (*n* = 5, 8%). When looking more into the methodology of quantitative studies, cross-sectional surveys were the most common (*n *= 16, 25%), followed by quasi-experimental studies pre-post design (*n* = 9, 14%), implementation studies (*n* = 7, 11%), cohort studies (*n* = 6, 10%) and randomized control trials (*n *= 5, 8%). Other quantitative designs such as comparison quasi experimental studies, case–control studies and geospatial analysis were less frequent (*n* ≤ 3). Qualitative studies all used semi-structured interviews to collect their data. Most of the mixed-methods studies were implementation studies (*n* = 4, 6%). Out of the 5 review articles (8%), 2 were systematic reviews (3%) and 3 were narrative reviews (5%). The objectives and outcomes of various studies differed greatly. Almost a third of the studies evaluated the vaccination uptake generated by different interventions in community pharmacies (*n* = 20, 32%).

The influenza vaccine was reported in almost half of the studies (*n *= 29, 46%). Herpes zoster, pneumococcal and human papilloma virus vaccines were each discussed in 14 studies (*n *= 22%), followed by tetanus-pertussis-diphtheria (*n *= 6, 10%) and travel vaccines (*n *= 2, 3%). Other vaccines figured in lower frequencies such as meningococcal vaccines, hepatitis A and B, measles-mumps-rubella or other children’s vaccinations (*n* ≤ 2). Thirteen studies investigated more than one vaccine at a time (21%). All but one combined the influenza vaccine with one or many other vaccines (*n* = 12, 19%). The combinations were influenza-pneumococcal (*n* = 4, 6%), influenza-pneumococcal-herpes zoster (*n* = 2, 3%), influenza-pertussis (*n *= 1, 2%) or a combination of more than 3 vaccines (*n* = 6, 10%).

### Vulnerability categories

We divided the various vulnerable populations into 5 categories of vulnerability: lifecycle vulnerabilities (*n* = 48, 76%), clinical factors (*n* = 18, 29%), socio-economical determinants (*n* = 16, 25%), geographical vulnerabilities (*n* = 7, 11%) and others (*n* = 6, 10%) (Table 3). A total of 22 articles combined more than one vulnerability category (35%).

**Table 3:** Frequency of vulnerability characteristics

Vulnerability Characteristics	Frequency	% (*n*=63)
**Lifecycle**		
Elderly	25	39.7%
Adolescent	12	19.0%
Pregnancy	4	6.3%
Women of childbearing age	3	4.8%
Parents of children	2	3.2%
Children	2	3.2%
**Clinical Factors**		
Combination of chronic conditions and/or immunodepression	9	14.3%
Pulmonary condition	4	6.3%
Diabetes	3	4.8%
Cardiac condition	1	1.6%
Cancer	1	1.6%
**Socio-Economic Determinants**		
Race	8	12.7%
Income	7	11.1%
Education	3	4.8%
**Geographical Factors**		
Geographical	9	14.3%
**Other**		
Occupation	3	4.8%
Lifestyle	2	3.2%
Incompleted vaccination	2	3.2%

First, within the lifecycle category, age-related criteria were the most prevalent such as being elderly (*n* = 25, 40%), adolescent (n = 12, 19%), of childbearing age (*n* = 3, 5%) or being a child (*n *= 2, 3%). Other subcategories within the lifecycle category include vulnerabilities around pregnancy and parenthood such as pregnant women (*n* = 4, 6%) and parents of children (*n* = 2, 3%).

Second, the clinical factors category regrouped a wide range of illnesses that increase the risk for complications such as pulmonary conditions (*n* = 4, 6%), diabetes (*n* = 3, 5%), cancer (*n* = 1, 2%), cardiovascular disease (*n* = 1, 2%) or a combination of at-risk illnesses or an immunocompromised status (*n*= 9, 14%). Illness status was identified via medical databases, insurance databases, pharmacy databases and self-reported medical history. One study defined its vulnerable population solely by the pharmacological profile by including patients that take more than 3 chronic medications [48]. One study also studied vaccination outcomes within a chronic condition management program [36].

Third, in the socio-economic determinants category, vulnerability is targeted through race (*n* = 8, 13%), income (*n* = 6, 10%) and education (*n* = 3, 5%). In most race-based studies, race was used to differentiate the proportion of users that obtain their vaccination in a pharmacy versus a medical setting. Some articles segmenting the study population with income focused on insurance status such as underserved adults (*n* = 2, 3%) [73, 83] or Medicaid beneficiaries (*n* = 2, 3%) [28, 35].

Fourth, geographical vulnerabilities were most often defined by contrasting rural and urban residence localization (*n* = 4, 6%). Other studies used a more precise categorization linked to accessibility such as medically underserved areas (*n* = 2, 3%) or social determinants of health such as low socio-economic status area (*n* = 1, 2%), racially and ethnically segregated neighborhoods (*n* = 1, 2%). One study (2%) used the state of origin [26] to contrast states where pharmacists are able and unable to administer vaccines.

Finally, the last category includes other vulnerabilities that did not fit in the previous categories such as occupation (*n* = 3, 5%), lifestyle (*n* = 2, 3%) and individuals with incomplete vaccination status (*n* = 2, 3%). Groups included in the occupation subcategory were military personnel, healthcare workers and students. The studies in the lifestyle category discussed travellers going to high-risk destinations. It is important to note that a third of the articles (*n* = 23, 37%) combined two or more vulnerability categories. The most common combination was clinical factors and lifecycle vulnerabilities (*n* = 14, 22%).

### Vaccination barriers and facilitators

**Fig. 2:**
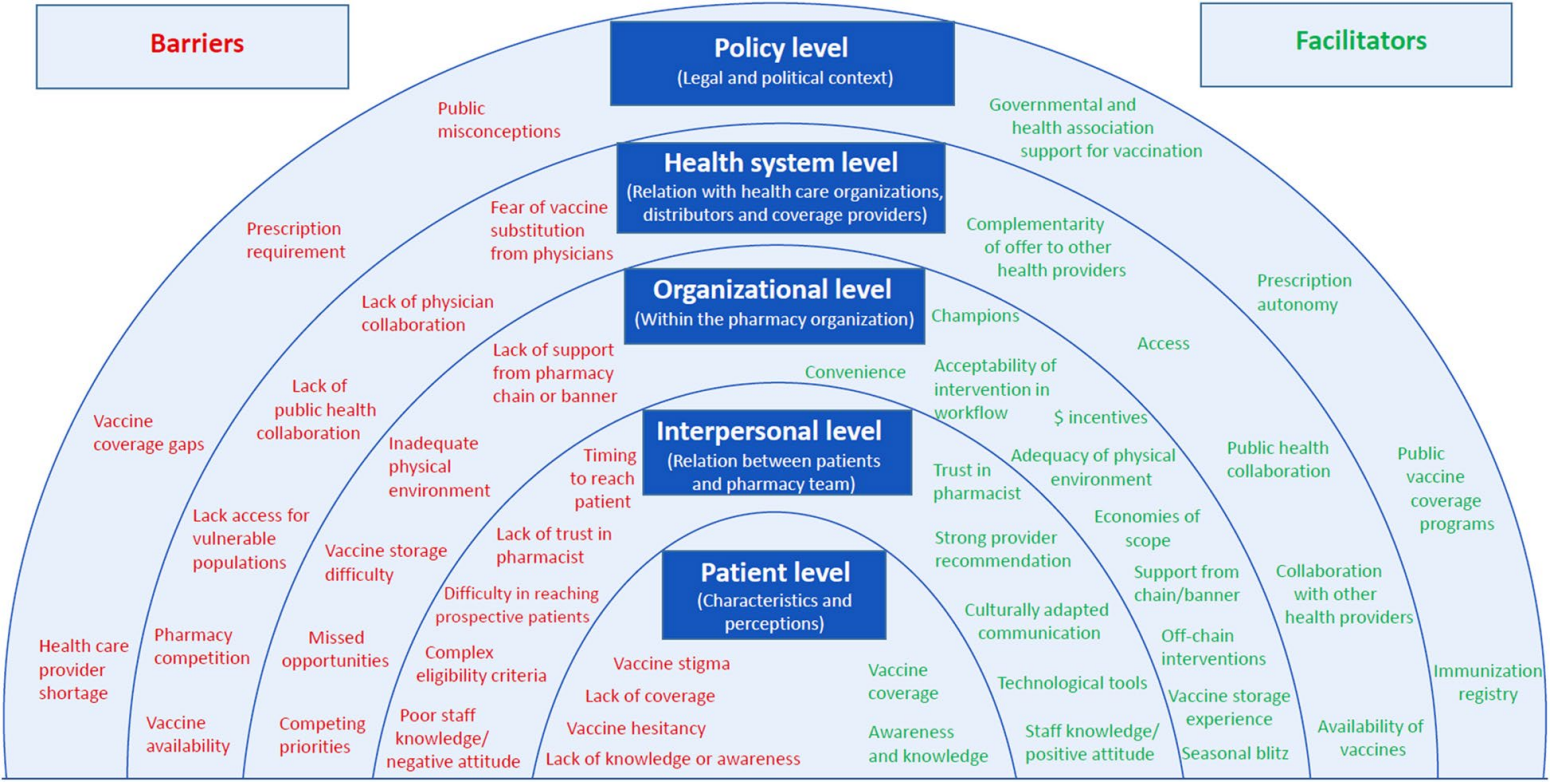
Vaccination Barriers and Facilitators in Community Pharmacy

Twenty-four barriers and 26 facilitators were compiled from the included articles (Table 4) and classified according to 5 levels (Fig. 2): 1) patient level (individual characteristics and perceptions), 2) interpersonal level (relationship between patients and pharmacy team members), 3) organizational level (factors within the pharmacy organization), 4) health system level (interaction between healthcare organizations, distributors and coverage providers) and 5) policy level (legal and political context) (Fig. 2).

**Table 4:** Frequency of barriers and facilitators

Barriers	Frequency	% of articles citing the barrier (*n* = 63)
**Patient Level**		
Lack of knowledge or awareness	15	23.8%
Lack of coverage	13	20.6%
Vaccine hesitancy	7	11.1%
Vaccine stigma	5	7.9%
**Interpersonal Level**		
Difficulty in reaching prospective patients	11	17.5%
Lack of trust in pharmacist	10	15.9%
Poor staff knowledge / negative attitude	10	15.9%
Complex eligibility criteria	8	12.7%
Timing to reach patient	5	7.9%
**Organizational Level**		
Competing priorities	15	23.8%
Missed opportunities	11	17.5%
Inadequate physical environment	4	6.3%
Lack of support from pharmacy banner of chain	3	4.8%
Vaccine storage difficulties	2	3.2%
**Health System Level**		
Lack of access for vulnerable populations	7	11.1%
Lack of public health collaboration	6	9.5%
Vaccine availability	6	9.5%
Lack of physician collaboration	5	7.9%
Fear of substitution from physicians	3	4.7%
Pharmacy competition	1	1.6%
**Policy Level**		
Prescription requirement	9	14.2%
Vaccine coverage gaps	5	7.9%
Healthcare provider shortage	2	3.2%
Public misconceptions	1	1.6%
**Patient Level**		
Awareness and knowledge	21	33.3%
Vaccine covered by insurance	8	12.6%
**Interpersonal Level**		
Strong provider recommendation	28	44.4%
Trust in pharmacist	10	15.9%
Technological tools for reminder and eligibility identification	12	19.0%
Strong knowledge from staff/positive attitude	11	17.5%
Culturally adapted communication	2	3.2%
**Organizational Level**		
Cost-saving/financial incentives	14	22.2%
Providing convenient modalities (e.g. extended-hours, walk-in)	12	19.0%
Acceptability of intervention in the workflow	8	12.7%
Adequacy of physical environment	4	6.3%
Economies of scope	2	3.2%
Support from pharmacy banner/chain	2	3.2%
Off work chain interventions	3	4.7%
Assigning a vaccination responsible	1	1.6%
Vaccine storage experience	1	1.6%
Seasonal blitz	1	1.6%
**Health System Level**		
Collaboration with other health providers	13	20.6%
Public health collaboration	11	17.5%
Accessibility	11	17.5%
Complementarity of offer to other health provider	2	3.2%
Availability of vaccines	2	3.2%
**Policy Level**		
Prescription autonomy	8	12.7%
Public vaccine coverage programs	5	7.9%
Immunization registry	4	6.3%
Governmental and health association support for vaccination	1	1.6%

Barriers were proportionally distributed amongst levels. Fifteen articles (24%) identified barriers originating from the patient’s lack of knowledge on vaccines or eligibility unawareness. Third-party reimbursement and the lack of coverage were also listed as a barrier in 13 articles (21%). Organizational obstacles such as other competing priorities (*n* = 15, 24%), missed opportunities (*n* = 11, 18%) and the requirement of a prescription from a physician (*n* = 9, 14%) were also mentioned.

Interestingly, interpersonal level facilitators were reported almost twice as often as other categories. Providing a strong recommendation for vaccination to a vulnerable patient was the most common facilitator and stated in 28 articles (44%). Other organizational helpers were utilizing a cost-saving or a promotional method that is tied to financial incentives (*n* = 14, 22%) or providing convenient modalities (walk-in, extended hours) to patients (*n* = 12, 19%). Many articles also stated health system facilitators such as collaboration with other providers (*n* = 13, 21%), collaboration with public health (*n* = 11, 17%) and the physical accessibility of pharmacies (*n* = 11, 17%).

### Targeting methods

Almost all of the included studies addressed interventions led by pharmacists (*n* = 60, 95%). The remaining 3 articles only involved pharmacy students (*n *= 2, 3%) and technicians (*n*= 1, 2%). Two studies described a clinical education program that was managed by pharmacy students, but under the supervision of pharmacists (3%) [33, 69]. One study reviewed the role of pharmacy technicians in gaping vaccination discrepancies [64]. Pharmacy technicians can contribute in bridging the discussion between pharmacy services and vaccination, keeping track of vaccination refusal and assisting in administrative tasks (documenting vaccines in the immunization records, collecting patient history, preparing the billing…). Eight articles (13%) also underlined the key role of pharmacy technicians in initiating the conversation about vaccination with eligible patients or referring to the pharmacist for further questions [29, 36, 42, 44, 64, 66, 69, 85].

Targeting methods can be defined as the tactics employed to identify, reach and distribute a service or a product to a specific group [86]. Twenty-three targeting methods were identified and divided between 3 categories: active promotion (14 strategies), passive promotion (6 strategies) and indirect promotion (3 strategies) (Table 5). Active promotional methods were diverse and involved the pharmacy team actively engaging and interacting with selected patients to promote vaccination [87]. The most common active promotion strategy was providing a strong recommendation for vaccination to patients (*n* = 25, 40%). Other strategies were distributing a bag stuffer or pamphlet (*n* = 17, 27%), initiation of a conversation on vaccine by a pharmacy team member (*n* = 8, 13%), sending a personalized letter (*n* = 8, 13%) or giving a personalized phone call to a vulnerable patient to promote a vaccine (*n* = 5, 8%). Some strategies were designed within the pharmacy workflow such as screening patients as they picked up the medication (*n* = 7, 11%) or programming a nudge in the pharmacy software notifying the pharmacists of an eligible patient (*n* = 4, 6%). Other strategies were better suited outside of the pharmacy workflow such as generating a list of eligible patients to offer them a vaccination appointment (*n* = 9, 14%), screening during another program such as a medication therapy review, a COPD medication review program or when receiving another vaccine (*n* = 6, 9.5%) or sending them an automated promotional phone call (*n* = 1, 2%). Some strategies aimed to educate patients through the distribution of an informational leaflet (*n* = 17, 27%) or providing educational group sessions to vulnerable patients (*n* = 3, 5%). Many articles reported collaboration with physicians (*n*= 13, 21%) such as recommending a vaccine to the patient’s physician, proactively asking them for a prescription or providing vaccination through a collective order. A collective order allows a health care professional that cannot prescribe vaccines to obtain a prescription signed by the responding physician without being evaluated by this physician [88]. One article addressed financial barriers by providing free influenza vaccine vouchers to underprivileged adults through community organisations (2%).

**Table 5:** Frequency of targeting strategies

Targeting Strategy	Frequency	% of articles citing the strategy (*n* = 63)
**Active Promotion**		
Total	125	-
Strong recommendation by a pharmacist	25	39.7%
Leaflet or bag stuffer	17	27.0%
Interprofessionnal collaboration (collective order)	13	20.6%
Generate lists of eligible patients from pharmacy software	9	14.3%
Conversation initated by pharmacy team	8	12.7%
Personalized letter	8	12.7%
Screening during workflow	7	11.1%
Screened during another pharmacy program	7	11.1%
Reminder call or note	7	11.1%
Personalized phone call	5	7.9%
Eligibility nudge within the prescription software	4	6.3%
Educational group sessions	3	4.8%
Financial aid for vaccine	2	3.2%
Automated phone call	1	1.6%
**Passive promotion**		
Total	35	-
Poster in pharmacy	16	25.4%
Advertising (TV, newspaper, radio)	6	9.5%
Promotion through other health care professional	4	6.3%
Convenient modalities (walk-in/extended hours)	4	6.3%
Social media advertising	3	4.8%
Word-of-mouth	2	3.2%
**Indirect promotion**		
Total	12	-
Staff training	8	12.7%
Culturally adapted communication	3	3.2%
Customized prescription pads	1	1.6%

Passive promotion strategies reach patients through smart positioning, media presence or a third party that does not directly generate an interaction with the pharmacy [87]. Within this category, we found the use of classic promotional methods such as a poster in pharmacies describing vaccination services, newspaper, TV and radio advertising (*n* = 6, 9.5%). Some articles reported promotional strategies using social media marketing (*n* = 3, 5%). Promotion was also done through word-of-mouth by patients and staff (*n* = 2, 3%) as well as through neighborhood health professionals (*n* = 4, 6%). Pharmacies also provide convenient modalities for vaccination such as walk-in and extended hours especially during mass influenza campaigns (*n* = 4, 6%).

Finally, some strategies were identified as indirect since they targeted the pharmacy staff instead of the patients or the vaccination process. Staff training (*n* = 8, 13%) was listed as an efficient method to make the pharmacy staff vaccine ambassadors. Teachings included improving knowledge on vaccines, providing assertive communication training and vaccine process training. Ensuring a culturally relevant communication (*n* = 3, 5%) was a way to improve how the message is perceived by the population. Finally, one article mentioned the use of customized prescription pads (2%) to facilitate the integration of vaccination within the pharmacy workflow.

**Fig. 3:**
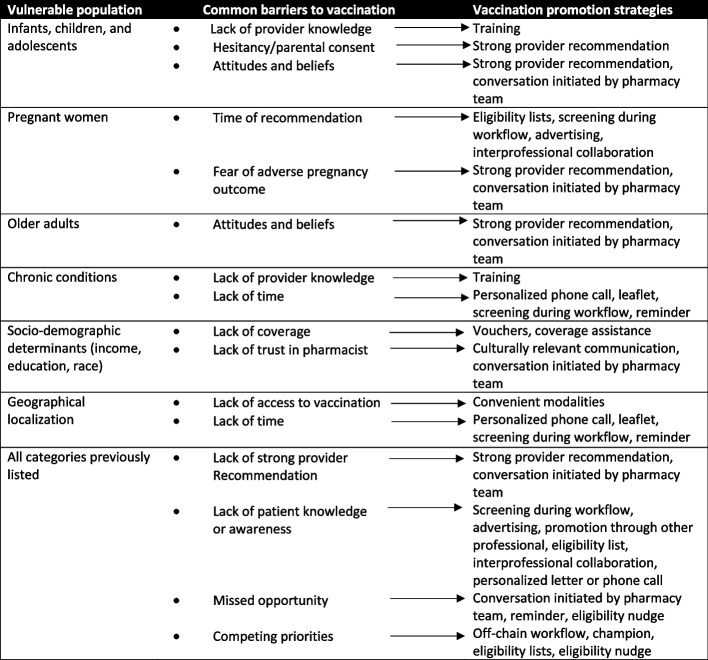
Promotion Strategies to Overcome Specific Barriers of Key Vulnerable Groups

Drawing from the previous data, we synthesized the barriers and promotional strategies to help pharmacists overcome vaccination challenges. We associated each vulnerable population to the common barriers identified in the included articles (Fig. 3). Those barriers were then linked to vaccination promotion strategies. We will first look at barriers and strategies that concern specific vulnerable populations. Pharmacists presented knowledge gaps with vaccines addressed to children and individuals with chronic conditions [48, 89]. These can be addressed through training on these specific populations [75]. Vaccine hesitancy, negative attitudes and personal beliefs against vaccines require time and an understanding the patient’s viewpoint [33]. A conversation between the pharmacist and the patient gives an opportunity to correct misconceptions, provide a strong reference for vaccination and call for action [34]. Timing issues such as not reaching pregnant women during their 3^rd^ trimester to offer pertussis vaccination [42] can be addressed by carefully monitoring the pharmacy’s population for vaccine eligibility through list generation or screening candidates during the workflow [44]. For patients that lack time to discuss or obtain a vaccine, pharmacists may rely on advertising and interprofessional collaboration to encourage patients to contact the pharmacy at a more convenient time for them [23]. Barriers to social-demographic determinants such as lack of coverage may be dealt with through facilitating reimbursement procedures with insurance [67] or through offering vouchers [45]. Trust may also be reinforced through relationship building with the pharmacy team and culturally relevant communication [28, 42, 64]. Providing convenient modalities for vaccination through walk-in or extended opening hours may reduce accessibility constraints that are frequent in rural areas [53, 73].

We identified 4 barriers that were common to every vulnerable category. The lack of a strong provider reference can be addressed by better linking patients to pharmacists through conversation initiation by the pharmacy team. Pharmacists and their team may overcome the lack of awareness to vaccines from vulnerable populations by actively screening patients in the workflow [44, 51], soliciting other health professional [50] or creating a list of eligible patients and contacting them through a letter or a phone call [36, 39]. To reduce missed opportunity, vaccination promotion should be discussed as a pharmacy team and involve every employee [42, 64, 66, 85]. A reminder system should be planned to reduce missed appointments and opportunities through notes in the file or nudges [37, 89]. An effective way to address competing priorities within the busy workflow is to move the workload outside of the regular distribution activities [30]. Designating a champion or key tasks to specific employees such as listing the eligible patients can help keep focus on vaccination through pharmacy activities [71].

## Discussion

This scoping review identifies a wide variety of studies targeting different populations considered as vulnerable by community pharmacists. Vaccinating vulnerable communities is dominantly studied in the United States where health discrepancy between race, economic status and geographical location are wide [4]. American pharmacists also benefit from decades of expanded scope of practice [90] which correlates with the wide body of articles published after 2014 (*n* = 44, 69.8%). We suspect that other regions of the world were underrepresented due to the language inclusion criteria and since pharmacists are predominantly involved in medication dispensing activities rather than clinical activities such as vaccination.

Vaccination has been a traditional activity of public health instances and pharmacists feel pressured to justify their value as efficient immunizers [10]. This has been observed in our review as more than a third of the studies have evaluated the vaccination uptake of pharmacists’ led interventions (*n* = 20, 31%). Qualitative and mixed-method studies provided a rich understanding of the dynamic of vaccination within the dispensing-centered mindset of pharmacies.

### The challenges of defining vulnerable communities

Pharmacists and their team target vulnerable communities in the included studies mainly based on life cycle criteria and clinical factors. They rely on the information that is available to them to assess eligibility. Age remains the most convenient method to target individuals but may oversimplify the rationale on risk prevention. On one hand, age provides a good statistical predictor of developing an illness such as influenza or pneumonia complication [91, 92]. Therefore, it appears fair to allocate more resources to better protect elderly populations. On the other hand, age may be a flawed indicator as life expectancy varies according to geographical localization or socio-economic determinants. Indeed, the gap in life expectancy varies according to income [93], education [94] and race [95] in the US. Disparities in life expectancy between rural and urban areas is however growing in the last 20 years and is attributable to cardiovascular and drug-overdose death [96]. Deciding on a cut-off to recommend a vaccine becomes a difficult exercise as years saved vary greatly according to the circumstances of each individual. Moreover, geriatric medicine is moving towards frailty score rather than age as means to aid in clinical decisions [97]. Many frailty scales provide a more detailed understanding of life expectancy or risk of complications, but have not been used in the field of vaccination.

Elderly people are also affected by the immunosenescence phenomenon which can be described as the waning of innate and cellular immunity [98]. The capacity to generate immunity is also affected by the clinical profile of a person. Some chronic diseases such as depression, cardiovascular diseases or conditions such as malnutrition, femur fracture or stress may decrease our capacity to generate immunity for a certain period of time [98–100]. Vaccinating while younger or prior to developing stress inducing conditions may be advantageous. Although scientific evidence on vaccination is complex, generating vaccination guidelines must remain simple for clinicians and easy to communicate to the public.

The list of chronic conditions affecting patients is not always easy to obtain in the community setting as diagnoses are seldom shared with the pharmacist. Pharmacists document in the patient’s pharmacological profile according to patients’ self-reported illnesses or by inference based on the patient’s medication. This process remains imperfect. One study directly used the number of medications as a mean to identify at-risk patients [79]. Correlating the number of medications provides a flawed view of vulnerability as some conditions such as single pathology like diabetes may require a combination of four or more oral treatments, while several other conditions may be targeted by a single tablet that contains a combination of drugs (e.g. antihypertensive and cholesterol lowering). Technological advancements and better diagnosis sharing between health professionals are ways to spend less on assessing a patient’s eligibility and more on promoting vaccination. As examples, suggestions range from a universal vaccine registry, to sharing the accesses to the pharmacological and medical file, to simplifying the eligibility criteria [50, 53, 63].

Other vulnerable groups provide their own targeting challenges. Considering that nearly half of all pregnancies in the US are unplanned [101], efforts to ensure adequate vaccination during pregnancy should be extended to all women of childbearing age. Prevention is however a wide concept, and the definition of at-risk groups widens as we discover additional risk factors. More and more, asymptomatic individuals with risk factors are treated with pharmacological drugs such as in hypertension or dyslipidemia which modifies our conception of health and sickness. Vaccines are also preventive medicines. In many jurisdictions, pharmacists are not able to actively participate in the preventions recommended in pregnancy as they cannot prescribe or administer vaccines against pertussis or other conditions within the regular vaccination calendar. Similarly, adolescents are the subject of many studies in our scoping review and the challenges rely on communication difficulty and patient unawareness of vaccination needs [33, 68]. Having a dual audience, both adolescents and their parents, confronts stereotypes and perceptions on sexuality which pharmacists and their team may feel uncomfortable to address. The timing to receive the vaccine does not always correlate with the optimal time to influence parental decision. More opportunities to discuss vaccination earlier on during childhood and schooling are necessary to increase vaccination uptake in adolescent and pregnant populations.

Social determinants of health and geographical factors are less frequently used to target vulnerable populations according to our results. From a pharmacy perspective, data on education levels and income are not readily available during workflow operations, which makes targeting for these vulnerabilities difficult. Conducting studies on adherents of an insurance program such as Medicaid [28, 35] appears to be the simplest way to study income disparities. Few studies attempt to target other individuals in precarious financial situations such as uninsured adults that do not qualify for Medicaid or underinsured students. Limited solutions are identified to overcome uninsured individuals. Addressing cost is one way to encourage vaccination by providing free vouchers to uninsured patients [45]. The cost of the program were assumed entirely by the pharmacy chain as part of a corporate social responsibility strategy, providing benefits to public health and promoting pharmacies as healthcare establishments [45]. Patient targeting was done with the help of community organizations and required readjustments on the 2nd year as redemption of the voucher was low (52% in 2015/2016 vs 87% in 2016/2017) [45]. Alternatively, many studies focus on access barriers to vaccination as less wealthy clienteles often require more flexible times and convenient modalities to access services. Vulnerability characteristics beyond age and chronic condition are therefore seldom integrated into targeting practices which shows a narrow understanding of vaccine disparity determinants.

### The forgotten groups

It is worth mentioning the absence of other marginalized communities from the scoping review, such as gender, sexual orientation and other marginalized communities. Females were targeted in studies that discussed vaccines specific to pregnancy or adolescence, which aligns with specific vaccine indications. However, no studies designed interventions to minimize vaccination discrepancy between men and women. Indeed, females are 42% more likely to receive an influenza vaccine then males when adjusted for common confounding factors [102]. Vaccine response also varies according to gender. When vaccinated against influenza, elderly women displayed greater humoral response against common flu lineage than elderly men, supposing a greater protection [103]. We must therefore understand that vulnerability goes beyond the mere expression of biological characteristics; we can seek answers in the structural construction of inequalities between groups.

Although specific LGBTQ key words were included in our search, no studies targeting this marginalized community came out. Men who have sex with men are disproportionately at risk of sexual transmitted disease which makes them candidates for Hepatitis B and HPV vaccines [104, 105]. Vaccines such as HPV address a sensitive topic, and pharmacists express discomfort discussing sexual health matters in a pharmacy setting [68]. Even though pharmacists are accessible health professionals, LGBTQ communities are reluctant to divulge their orientation due to fear of judgment or lack of confidentiality [106]. More efforts are needed to make pharmacies an inclusive and safe environment. Positive actions towards inclusivity can be displayed through offering information pamphlets specific to LGBTQ stakes, communicating with inclusive vocabulary or showing support to the community [107].

Other hard-to-reach communities such as injectable drug users, patients receiving an opioid agonist therapy or homeless people are at higher risk of infection and thus good candidates for vaccination [108]. These populations are often stigmatised by many societal institutions and are less inclined to be offered and receive preventative services. Community pharmacies may have better opportunities than other health care entities to build a trusting relation with these individuals due to easy access. Some opportunities may present themselves during dispensing activities for example when distributing clean needles, naloxone kits or other medications.

### Facilitating vaccine promotion

Barriers identified regarding vulnerable groups were consistent within the literature in other contexts than in pharmacy. In the context of pregnancy, two such examples are the fear of adverse pregnancy outcome and the failure to recommend vaccination [109]. The knowledge gap from healthcare providers is listed as an important barrier [110, 111]. Patients unaware that a vaccine is recommended often wanted to contact their family physician before obtaining the vaccine, which delays vaccination. This reason was cited as a common barrier in the studies we reviewed and increased the risk of not pursuing the vaccination [44]. Although pharmacists are trustworthy professionals, they may be competing with the existing relationship that patients build with other health professionals [69]. The requirement for a prescription in many jurisdictions also contributed as a supplemental barrier that made vaccination less convenient in a pharmacy than at the physician’s office [38, 44]. Interprofessional collaborations remain a well noted facilitator in vaccination [21] and healthcare professionals should unite their voice to carry out a cohesive message supporting vaccination.

In the past decade, community pharmacies are transitioning from a dispensing business model to increased clinical services [112]. Although role expansion is stimulating, pharmacists are trained to consider the medication profile as a primary source of information rather than contextual and social vulnerabilities. Chronic conditions become a proxy to vulnerability at large and may simplify the interrelations between illnesses and other social determinants of health that impact access to vaccination. Organizational barriers are frequently reported and center around missed opportunities and competing priorities. Pharmacists have traditionally been reactive vaccinators [21]. This can be attributed to the fact that routine assessment of vaccination status was never a responsibility attributed to pharmacists up until recently. Although active promotional strategies were more frequently cited than passive strategies in the peer-reviewed literature, we have doubts that this reflects the pharmacy practice in the real world. Proactivity in pharmacy is often expressed through the display of posters and handing out informational leaflets [42] which alone are poor methods to impact behavioral change [113]. Many pharmacies may also not establish a formal targeting plan. This may result in voluntarily or involuntarily favouring privileged clienteles. A dispensing centered mentality pushes pharmacists towards reacting to patient’s demand instead of acting proactively. Pharmacies often rely on a ‘’first come, first serve’’ prioritization strategy which accentuates vaccine discrepancies of vulnerable communities [114]. Technology should be utilized to assist vaccine operations such as booking appointments and accessing vaccination history. Pharmacies should also make their dispensing operation more efficient to free time for value-added activities such as targeting at-risk patients. Pharmacists can plan vaccination outside of the pharmacy workflow and solicit the help of pharmacy technicians to identify eligible patients and initiate the conversation on vaccines [30]. As vaccine hesitancy is a growing concern, health professionals need to invest time and energy to educate patients on vaccines safety and effectiveness [115].

Promotional efforts made by pharmacists are complementary to governmental, public health and pharmacy chains advertising. The effect of different communications according to race on attitudes towards pneumococcal vaccination was investigated [43]. Non-White adults were less likely to follow medical recommendations and more likely to desire vaccination when the message combined duty to family and friends, fatality or safety [43]. More research is therefore necessary to better understand the core values of different populations and investigate how they were made “vulnerable” to adapt how we reach these patients. Our review highlights the importance of a strong recommendation for vaccination by pharmacists and was confirmed in a recent review on vaccine acceptance [43]. Although the efficiency of many strategies lacks proof, utilizing a combination of different strategies and providing a strong recommendation from a health provider are known as the most effective ways to encourage vaccination [116]. As pharmacists build strong relationships with their clientele, they must mobilize their team to create opportunities for a tailored conversation about vaccines and utilize their position as one of the most accessible healthcare professionals.

### Limitations

First, our search strategy included 2 databases and may have overlooked some articles in the literature. Other databases could have been included such as Scopus or Web of science, but they usually provide similar results. References from the included studies could have been reviewed to find additional relevant publications. Second, our review only included published articles from the literature and did not include gray literature which may also hold valuable information regarding targeting practices in pharmacy. Third, our study targeted vulnerable communities from the perspective of pharmacies. Barriers to promoting vaccination and promotional methods are therefore subject to a selection bias within the different efforts carried out by public health instances. The search was also performed before the COVID-19 mass campaigns which allowed pharmacists to take part in vaccination efforts. Barriers and facilitators may therefore be different after the COVID-19 pandemic response. Fourth, our sample agglomerated heterogenous articles in terms of methodology, main topic, and the vaccines they discussed. Some conclusions must therefore be interpreted with caution as the reality of different vulnerable groups and vaccination may vary.

## Conclusion

Throughout the last two decades, pharmacists have increasingly been involved in vaccination activities. Our scoping review highlights the use lifecycle and clinical dimensions to define vulnerability and to target patients identified as vulnerable, at the expense of narrowing down the definition of vulnerability and its process. Social determinants of health such as one’s race, income and geographical situation are important contributors of vaccine inequality. Indeed, some marginalized groups are absent form the vaccine promotion literature in pharmacy such as intravenous drug users, the LGBTQ community and homeless people. Targeting such communities requires an intricate knowledge of the barriers to vaccination, that range from a lack of access, awareness of vaccination, misconceptions to financial obstacles. A variety of active, passive, and indirect targeting methods were used by pharmacists through various vaccination initiatives. We linked them to the main barriers experienced by different groups. Pharmacists are trusted health professionals and as valuable contributors to public health goals; it is their responsibilities to include vulnerability concepts into their targeting initiatives integrate.

This review should inspire researchers to further expand our knowledge on how to define vulnerable communities in vaccination to better serve them. A conversation between public health and community pharmacies representative is much needed in this respect. Although studies center around influenza vaccination, more research is needed to better understand the drivers and the facilitators to vaccination programs for other vaccine preventable diseases, including industry-based strategies. Vaccination organization varies according to pharmacies and jurisdiction and has repercussions on the clienteles targeted by pharmacists. A deeper understanding of how pharmacists interact and collaborate with different entities will also aid policy makers and public health representatives to better align incentives to desired outcomes. Improving stagnating vaccination rates requires a collaborative effort from all pharmacy employees as well as a continuous reflection exercise on the efforts made to attract underserved communities. Pharmacists can play an even greater role in vaccination through leveraging their position as accessible, competent, and trustworthy health professionals.

### Supplementary Information

**Additional file 1: Supplementary material 1.** Search strategy.

## Data Availability

All data generated or analysed during this study are included in this published article (and its supplementary information files).
