# Supplementary material for: Vaccine promotion strategies in community pharmacy addressing vulnerable populations: a scoping review
Source: BMC Public Health. 2023 Sep 23;23:1855. doi: 10.1186/s12889-023-16601-y (PMC10518112; doi:10.1186/s12889-023-16601-y)
Supplement: Supplementary file 1 — Additional file 1: Supplementary material 1. Search strategy. [file 12889_2023_16601_MOESM1_ESM.docx]

**Supplementary material 1 :** Search strategy

|  | **#** | **Searches** |
| --- | --- | --- |
| **Embase Search Strategy** | | |
|  | 1 | exp Vaccination/ or Vaccination Coverage/ or Vaccination Refusal/ or exp vaccine/ |
|  | 2 | (Vaccination? or Immuni#ation? or Vaccine?).tw. |
| A | 3 | 1 or 2 |
|  | 4 | exp "pharmacy (shop)"/ |
|  | 5 | ((Community or retail or chain) adj Pharmac*).tw. |
|  | 6 | ((Pharmac* adj (Service* or care or practitioner?)) and communit*).tw. |
|  | 7 | (pharmacist? adj4 communit*).tw. |
|  | 8 | pharmacy shop?.tw. |
| B | 9 | 4 or 5 or 6 or 7 or 8 |
|  | 10 | exp vulnerable populations/ or high risk population/ or lgbtqia+ people/ or exp lgbt people/ or "sexual and gender minority"/ |
|  | 11 | ((Vulnerable or Sensitive or high risk or Underserved or under-served or homeless or LGBTQ* or gay? or lesbian?) adj (Population? or Patient? or client* or person? or senior? or group?)).tw. |
| C | 12 | 10 or 11 |
| ABC | 13 | 3 and 9 and 12 |
| ABC limited | 14 | limit 13 to (embase and (english or french or spanish)) |
| AB not ABC | 15 | (3 and 9) not 13 |
| AB not ABC, limited | 16 | limit 15 to (embase and (english or french or spanish)) |
| **MEDLINE Search Strategy** | | |
|  | 1 | exp Vaccination/ or Vaccination Coverage/ or Vaccination Refusal/ or Immunization Programs/ or exp Vaccines/ |
|  | 2 | (Vaccination? or Immuni#ation? or Vaccine?).tw. |
| A | 3 | 1 or 2 |
|  | 4 | Community Pharmacy Services/ |
|  | 5 | ((Community or retail or chain) adj Pharmac*).tw. |
|  | 6 | ((Pharmac* adj (Service* or care or practitioner?)) and communit*).tw. |
|  | 7 | (pharmacist? adj4 communit*).tw. |
|  | 8 | pharmacy shop?.tw. |
| B | 9 | 4 or 5 or 6 or 7 or 8 |
|  | 10 | Vulnerable Populations/ |
|  | 11 | ((Vulnerable or Sensitive or Underserved or under-served or homeless or LGBTQ* or gay? or lesbian?) adj (Population? or Patient? or client* or person? or senior? or group?)).tw. |
| C | 12 | 10 or 11 |
| ABC | 13 | 3 and 9 and 12 |
| ABC limited | 14 | limit 13 to (english or french or spanish) |
| AB not ABC | 15 | (3 and 9) not 13 |
| AB not ABC, limited | 16 | limit 15 to (english or french or spanish) |
